# Geographic variations, temporal trends, and equity in healthcare resource allocation in China, 2010–21

**DOI:** 10.7189/jogh.15.04008

**Published:** 2025-01-17

**Authors:** Shaohua Yin, Zhenlin Liu, Sujuan Yu, Ying Li, Ji An, Dong Wang, Hongjia Yan, Ying Xiao, Feng Xu, Yun Tian, Xiaoxiao Luan

**Affiliations:** 1 Department of Medical Engineering, Peking University Third Hospital, Beijing, China; 2 Department of Orthopaedics, Peking University Third Hospital, Beijing, China

## Abstract

**Background:**

Inequity in healthcare resources has been identified as a global public health priority, yet the geographic variations and temporal trends in distribution and inequity in China remain unclear. We aimed to investigate these variations and temporal trends in healthcare resources and evaluate inequity in healthcare resource allocation in China.

**Methods:**

In this nationwide descriptive study, we used provincial-level data on healthcare infrastructure, human, and service resources from 31 provinces of mainland China, publicly released by the National Health Commission of China between 2010–21. We assessed the spatial autocorrelation of healthcare infrastructure, human, and service resources using Moran’s I index, and identified spatial clusters of resource allocation. We evaluated the equity in healthcare resource allocation using the Lorenz curve, Gini coefficient, and Theil index by population and geographic dimensions.

**Results:**

Between 2010–21, the density of healthcare infrastructure and human resources in China increased, with the average stay decreasing from 10.5 to 9.2 days. There were substantial regional disparities, with higher resource density exhibited in eastern regions compared to western regions. Spatial autocorrelation was more pronounced for the density of practising (assistant) physicians (Moran’s I = 0.465; *P* < 0.001), practising physicians (Moran’s I = 0.351; *P* < 0.001), and bed occupancy rate (Moran’s I = 0.256; *P* < 0.001), with significant geographic clusters of resource allocation. Lorenz curves showed that healthcare resource allocation was closer to the absolute equity by population but not geographic dimension, with Gini coefficients indicating severe inequity (>0.6) by geographic dimension compared to perfect equity (<0.2) by population dimension. Intraregional Theil index by population was higher than the inter-regional index, with contribution rates exceeding 60%.

**Conclusions:**

Per capita access to healthcare resources in China has improved, but significant geographic variations and clustering exist, particularly with higher resource density in eastern regions. While resource allocation by population showed better equity than by geographic area, substantial intra-regional disparities highlight the need for targeted strategies to enhance equitable distribution, particularly in the western regions.

Inequity in healthcare resources remains a critical global public health challenge, associated with increased mortality, lacking confidence in healthcare systems, and economic side effects [1,2]. Despite improvements in global health, especially in low- and middle-income countries (LMICs), substantial disparities persist in the distribution of human, healthcare service, and infrastructure resources between and within countries [3–5], suggesting a major challenge to achieving Sustainable Development Goals [1,2]. For example, the Global Burden of Disease 2019 study showed that Sweden has 696.1 healthcare staff per 10 000 population, while Ethiopia has only 13.9 and Guinea 15.1 staff per 10 000 population [2]. The Lancet Oncology Commission on Global Cancer Surgery projected that by 2030, only 25% of the 21.6 million cancer patients needing surgical intervention will have access to safe, affordable, or timely surgery, particularly in LMICs [6,7]. The International Atomic Energy Agency reported that high-income countries provide one radiotherapy device per 250 000 population, compared to one per seven million in developing countries [8].

Driven by economic globalisation and China’s robust economic growth, China has undergone rapid healthcare advancements over the past 15 years [9]. In 2009, China initiated a new round of healthcare reforms aimed at achieving universal health coverage by 2020 [10], expanding social health insurance and strengthening primary healthcare capacity while narrowing urban-rural disparity in accessibility of medical resources [11,12]. Subsequent initiatives, including the Healthy China 2030 Plan and the Healthy China Action (2019–30), prioritise optimising health services, improving healthcare coverage, and advancing high-quality universal health coverage [13,14].

Although these economic and health policies have yielded considerable improvements in the healthcare sector, the geographic variation and temporal trend in healthcare resource equity remain unclear. A nationwide longitudinal study in mainland China showed a substantial rise in the number of general physicians per 10 000 population, increasing from 0.81 in 2012 to 3.08 per 10 000 population in 2021 [15]. Similar findings were observed in studies from Guangdong between 2017–21 [16] and Western China between 2014–18, which examined physicians and nurses [17]. Furthermore, population-based cohort studies in China found a reduction in length of stay, with pneumonia hospitalisations reflecting a –1.0% annualised change from 2009 to 2017 [18], and avoidable hospitalisations from chronic diseases decreased from –5.2% to –10.5% between 2015–18 [19]. However, these studies had limitations, including limited indicators, insufficient exploration of the sources of variation, and the use of outdated data.

Using data from China Health Statistics Yearbooks for 31 provinces in mainland China between 2010–21, we aimed to assess geographic variations and temporal trends in healthcare resource allocation and to investigate its inequity by population and geographic dimensions.

## METHODS

### Data sources

Our primary data source was the National Health Statistics Yearbook, an annual compilation released by the National Health Commission of China since 2000 [20]. The yearbook provides provincial-level indicators related to healthcare resources and services and the health status of residents, including data for all populations, healthcare institutions and provinces, aiming to address the progress, achievements, and experiences in China’s health sector. Healthcare data from different institutions such as hospitals, primary healthcare facilities, and specialised public healthcare institutions in every county, township, and village are collected and aggregated annually through the National Health Surveillance Information Reporting System. The aggregated county-level data were reviewed and validated by trained staff and then reported to the city and provincial health commissions and then to the National Health Commission of China by the online reporting system.

### Data extraction and cleaning

We selected a series of healthcare resource indicators to assess the healthcare resource allocation in China based on a literature review [2,9,15,16,21,22] and expert consultation. We extracted yearly data on healthcare infrastructure resources, including the number of healthcare institutions, primary healthcare institutions, and equipment with a value of ≥CNY 10 000, and healthcare human resources, including both total and specific healthcare technicians (*e.g.* practising physicians, practising (assistant) physicians, registered nurses), and healthcare service resources including bed occupancy rate (calculated as the total actual occupied bed days divided by the total actual available bed days), average length of stay (calculated as the total number of bed occupation days of inpatients divided by the total number of inpatients), the number of outpatient visits in healthcare institutions, and the number of outpatient visits in primary healthcare institutions, from 2010 to 2021 at provincial level from National and Provincial Health Statistics Yearbooks (Table S1 in the **Online Supplementary Document**). We calculated the density of healthcare infrastructure and human resources as the number of healthcare institutions, equipment, and technicians per total population, expressing these ratios per 1000 and 10 000 individuals, as appropriate. Additionally, we extracted annual data on maternal mortality, perinatal mortality, and average life expectancy from the China Social Statistical Yearbook. Information on the total population and geographic area at the provincial level was obtained from the China Statistics Yearbooks. According to China’s administrative division, the 31 provinces in mainland China were categorised into eastern, central, and western regions, indicating different geographic locations. Data collection followed standardised protocols, and aggregated data was cross-validated with data from other reliable sources to ensure accuracy [9,16,21]. Records for healthcare resources, including those for equipment with a value of ≥CNY 10 000 with missing data, were included in the analysis as a full dataset.

### Statistical analysis

#### Spatial autocorrelation analyses

We performed the spatial autocorrelation analyses using the global and local Moran’s I indices to evaluate the clustered or dispersed patterns of healthcare resources within the study area. We calculated the global Moran’s I index, ranging from –1 to 1, to assess global spatial autocorrelation within the study area, indicating the spatial distribution pattern of resources. Positive values indicate that nearby regions have similar resource levels (spatial clustering), while negative values indicate dissimilar resource levels (spatial dispersion). Values approaching zero suggest a random spatial distribution. We calculated the local Moran’s I index [23] to determine local areas with particularly high or low resource levels relative to the mean and to identify clustered patterns, such as hotspots (high-resource areas surrounded by other high-resource areas), cold spots (low-resource areas surrounded by other low-resource areas), and dispersion (spatial outliers, areas where high-resource regions surrounded by low-resource regions or vice versa), and Monte Carlo simulations were used to determination of the statistical significance of spatial clustering patterns (Method S1 in the **Online Supplementary Document**) [24].

#### Lorenz curve, Gini coefficient, and Theil index

We used the Lorenz curve, Gini coefficient, and Theil index to assess the inequity in healthcare resource allocation among 31 provinces, considering population and geographic dimensions (Method S1 in the **Online Supplementary Document**). The Lorenz curve is a two-dimensional scatter plot with population size and geographic area ranking on the X-axis and the cumulative percentage of healthcare resources on the Y-axis. A diagonal line represents perfect equality, with greater deviation from this line indicating increased inequity [2,25]. The Gini coefficient quantifies the overall inequity as the ratio of the area between the Lorenz curve and the line of perfect equality to the total area below that line. A Gini coefficient of zero indicated perfect equality, while a coefficient of 1.0 indicated perfect inequity. Specifically, values <0.2 indicated perfect equality, 0.2–0.3 indicated relative equality, 0.3–0.4 indicated proper equality, 0.4–0.5 indicated relative inequity, and values >0.5 indicated severe inequity [22].

The Theil index, distinct from the Gini coefficient, allows us to decompose total provincial inequity into differences between regions and within regions to understand the sources of inequity [26]. It is calculated by the population size or geographic area of each province and healthcare resources compared to the total resources available. The index starts from zero, representing no inequity, with higher scores indicating increasing inequity with no upper limit [27].

We considered significant a two-sided *P*-value ≤0.05. We conducted statistical analyses using SAS, version 9.4 (SAS Institute Inc, Cary, NC, USA), and for geographic analyses, we used ArcGIS, version 10.2 (Esri, Redlands, CA, USA).

## RESULTS

### Temporal trends in healthcare resources in China

Between 2010–21, the number of healthcare institutions across 31 provinces in mainland China increased substantially from 936 927 to 1 030 935, corresponding to an increase in crude density from 6.99 to 7.30 institutions per 10 000 population. Among these, primary healthcare institutions were the predominant category, increasing from 901 709 in 2010 to 977 790 in 2021, with a density increase from 6.72 to 7.29 institutions per 10 000 population (**Table 1**). The density of equipment with a value of ≥CNY 10 000 rose from 1.71 to 7.54 per 1000 population. Additionally, the density of healthcare technicians showed a notable increase, rising from 4.38 to 7.96 per 1000 population. Moreover, both the bed occupancy rate and average length of stay decreased annually, from 86.7% to 74.6% and from 10.5 to 9.2 days, respectively. The number of outpatient visits to healthcare institutions in 2021 more than doubled in 2010, with a slight increase observed in outpatient visits to primary healthcare institutions (**Table 1**).

**Table 1:** The distribution of healthcare resources in mainland China from 2010 to 2021*

	Year											
**Resources**	**2010**	**2011**	**2012**	**2013**	**2014**	**2015**	**2016**	**2017**	**2018**	**2019**	**2020**	**2021**
Healthcare infrastructure resources												
*Healthcare institutions per 10 000 population*	6.99	7.07	6.99	7.13	7.13	7.11	7.06	7.05	7.10	7.15	7.24	7.30
*Primary healthcare institutions per 10 000 population*	6.72	6.85	6.81	6.83	6.84	6.87	6.91	6.96	7.04	7.12	7.23	7.29
*Equipment with a value of ≥CNY 10 000 per 1000 population†*	1.71	1.89	2.11	3.20	3.55	2.85	3.60	4.82	5.30	5.84	6.65	7.54
Healthcare human resources												
*Healthcare technicians per 1000 population*	4.38	4.60	4.91	5.27	5.51	5.79	6.07	6.42	6.78	7.20	7.56	7.96
*Practising (assistant) physicians per 1000 population*	1.80	1.83	1.92	2.04	2.10	2.20	2.29	2.42	2.57	2.74	2.89	3.04
*Practising physicians per 1000 population*	1.47	1.50	1.57	1.67	1.73	1.81	1.90	2.02	2.14	2.28	2.41	2.54
*Registered nurses per 1000 population*	1.53	1.66	1.84	2.04	2.18	2.34	2.52	2.72	2.92	3.15	3.33	3.55
Healthcare service resources												
*Beds occupancy rate (%)*	86.70	88.50	90.10	89.00	88.00	85.40	85.30	85.00	84.20	83.60	72.30	74.60
*Beds per 1000 population*	3.57	3.82	4.21	4.52	4.80	5.07	5.32	5.67	5.98	6.25	6.44	6.69
*Length of stay in days*	10.5	10.3	10.0	9.8	9.6	9.6	9.4	9.3	9.3	9.1	9.5	9.2
*Outpatient visits in healthcare institutions*	3 327 043 590	6 271 226 278	6 888 329 138	7 314 009 678	7 601 866 343	7 693 425 129	7 931 700 496	8 183 109 952	8 308 016 930	8 719 873 082	7 741 048 129	8 472 033 436
*Outpatient visits in primary healthcare institutions*	361 156	380 560	410 921	432 431	436 395	434 193	436 663	442 892	440 632	453 087	411 614	425 024

*The total distribution of healthcare resources was summarised using absolute numbers (outpatient visits in healthcare institutions, and outpatient visits in primary healthcare institutions) or ratios for continuous variables, as appropriate.

†Including 15 provinces across eastern (Beijing, Liaoning, Jiangsu, Fujian, Shandong, and Guangdong), central (Heilongjiang, Jiangxi, Hubei, and Hunan), and western regions (Inner Mongolia, Sichuan, Yunnan, Shaanxi, and Gansu) in mainland China.

### Geographic variations in healthcare resources

The allocation of healthcare resources in China showed substantial regional disparities (Table S2 in the **Online Supplementary Document**). For instance, in 2021, the density of healthcare institutions in the eastern region was 6.49 per 10 000 population, while it was 7.72 in the central region and 8.16 in the western region. The density of equipment with a value of ≥CNY 10 000 was 8.13 per 1000 population in the eastern region, 6.72 in the central region, and 7.16 in the western region. For healthcare human resources, the eastern region had higher densities of practising (assistant) physicians (3.19 per 1000 population) and practising physicians (2.73 per 1000 population), compared to 2.96 and 2.42 in the central region and 2.88 and 2.39 in the western region. In addition, the average length of stay varied slightly across regions, with 9.1 days in the eastern region, 9.5 in the central, and 9.2 in the western region.

**Figure 1:**
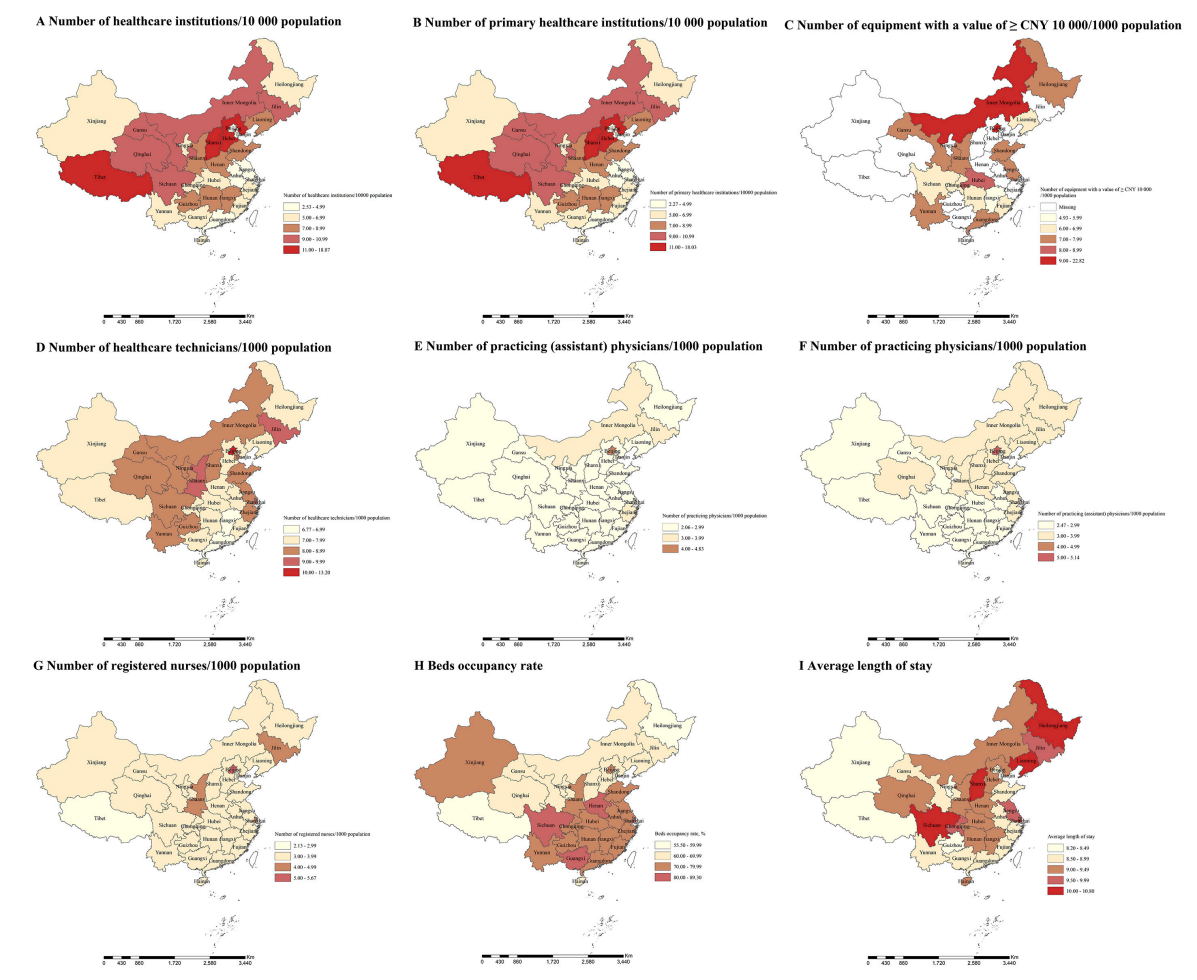
Distribution of healthcare resources in mainland China in 2021. **Panel A.** Distribution of the number of healthcare institutions per 10 000 population. **Panel B.** Distribution of the number of primary healthcare institutions per 10 000 population. **Panel C.** Distribution of the number of equipment with a value of ≥CNY 10 000 per 1000 population. **Panel D. **Distribution of the number of healthcare technicians per 1000 population. **Panel E. **Distribution of the number of practising (assistant) physicians per 1000 population. **Panel F. **Distribution of the number of practising physicians per 1000 population. **Panel G.** Distribution of the number of registered nurses per 1000 population. **Panel H. **Distribution of the bed occupancy rate.** Panel I. **Distribution of an average length of stay.

At the provincial level in 2021, the density of healthcare institutions ranged from 2.53 per 10 000 population in Shanghai to 18.87 in Tibet (**Figure 1**). Similarly, the density of primary healthcare institutions ranged from 2.27 in Shanghai to 18.03 in Tibet. Regarding equipment with a value of ≥CNY 10 000, the density ranged from 4.93 in Jiangxi to 22.82 per 1000 population in Beijing. For healthcare technicians, the density varied from 6.77 in Jiangxi to 13.20 per 1000 population in Beijing. Likewise, the density of practising (assistant) physicians ranged from 2.47 in Jiangxi to 5.14 per 1000 population in Beijing, and those of practising physicians ranged from 2.06 per 1000 population in Jiangxi to 4.83 in Beijing. Additionally, the density of registered nurses ranged from 2.13 per 1000 population in Tibet to 5.67 in Beijing. Bed occupancy rates showed substantial diversity, ranging from 55.5% in Heilongjiang to 89.3% in Shanghai in 2021. The average length of stay varied across provinces, ranging from 8.2 days in Tibet to 10.8 days in Heilongjiang (**Figure 1**). The geographic variation persisted after accounting for gross domestic product (GDP), the proportion of women, the urbanisation rate, the marriage rate, and the percentage of the population aged ≥15 years (Table S3 in the **Online Supplementary Document**). There was a significant relationship between healthcare resources and health outcomes, with maternal and perinatal mortality rates decreasing as healthcare resource density increased and average life expectancy rose (Figure S1–3 in the **Online Supplementary Document**).

### Spatial autocorrelation of healthcare resources

**Figure 2:**
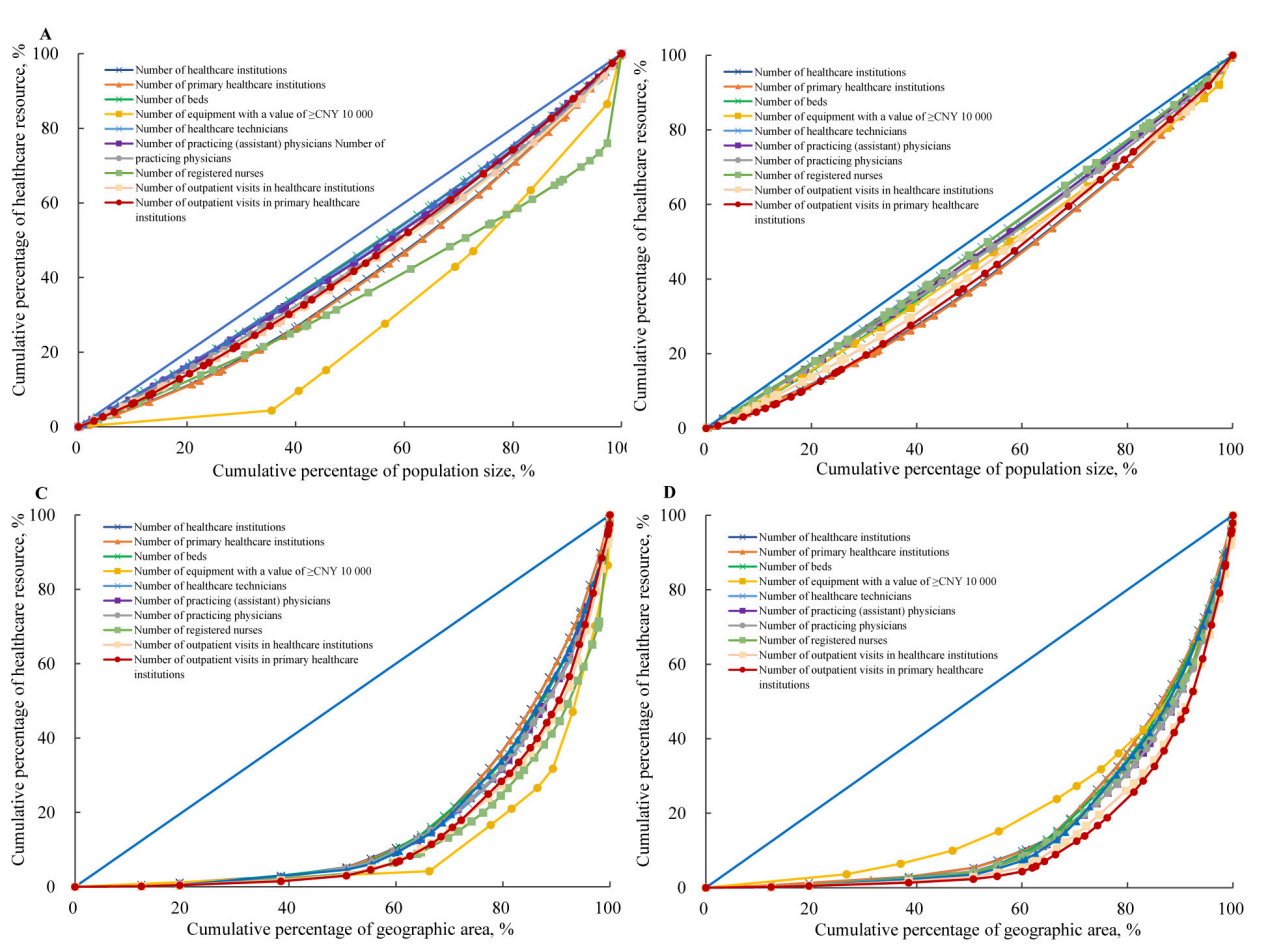
Lorentz curve of healthcare resources in mainland China between 2010–21. **Panel A. **Allocation by population in 2010. **Panel B. **Allocation by population in 2021. **Panel C.** Allocation by geographical area in 2010. **Panel D.** Allocation by geographical area in 2021.

In 2021, substantial geographic clustering for healthcare resources across China was observed (**Figure 2**; Figure S4 in the **Online Supplementary Document**). The global spatial autocorrelation analysis showed significant spatial clustering of provincial-level densities of practising (assistant) physicians (global Moran’s I = 0.465; *P* < 0.001), practising physicians (global Moran’s I = 0.351; *P* < 0.001), and bed occupancy rate (global Moran’s I = 0.256; *P* < 0.001) (Figure S4 in the **Online Supplementary Document**). The local spatial autocorrelation analysis showed a distinct geographic divide for high-density (hotspots) and low-density (cold spots) healthcare resources across 31 provinces (**Figure 2**; Table S4 in the **Online Supplementary Document**). For example, high densities of practising (assistant) physicians were concentrated in eastern regions (Beijing, Hebei, and Tianjin), and low densities were observed mostly in the western regions (Guizhou and Guangxi).

### Lorenz curves and Gini coefficients of healthcare resources

**Figure 3:**
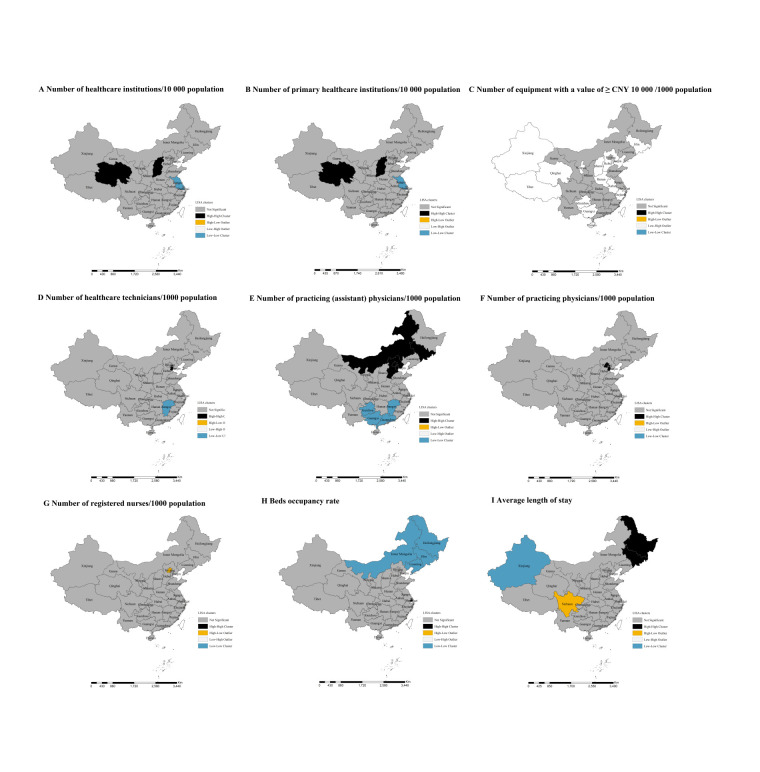
Cluster maps for healthcare resources in mainland China in 2021. **Panel A.** Spatial analysis for the number of healthcare institutions per 10 000 population. **Panel B.** Spatial analysis for the number of primary healthcare institutions per 10 000 population. **Panel C. **Spatial analysis for the number of equipment with a value of ≥CNY 10 000 per 1000 population. **Panel D. **Spatial analysis for the number of healthcare technicians per 1000 population. **Panel E. **Spatial analysis for the number of practising (assistant) physicians per 1000 population. **Panel F. **Spatial analysis for the number of practising physicians per 1000 population. **Panel G.** Spatial analysis for the number of registered nurses per 1000 population. **Panel H. **Spatial analysis for bed occupancy rate. **Panel I. **Spatial analysis for an average length of stay. High-high regions have adequate healthcare resources and are surrounded by other regions with adequate healthcare resources. Low-low regions have inadequate healthcare resources and are surrounded by other regions with inadequate healthcare resources. These regions contribute positively to spatial autocorrelation. High-low regions have adequate healthcare resources and are surrounded by regions with inadequate healthcare resources, whereas low-high regions have inadequate healthcare resources and are surrounded by regions with adequate healthcare resources. These regions contribute negatively to spatial autocorrelation.

The Lorenz curves by population dimension showed that healthcare resource allocation was closer to the absolute equity line between 2010–21, while the curves by geographic dimension were farthest from the absolute equity line (**Figure 3**). The temporal trends in Gini coefficients for both dimensions were stable or showed a slight negative linear trend for most healthcare resources, suggesting improved equity over time (**Table 2**). Similar results were observed for the difference between the 5th to 95th percentiles of healthcare resources (Table S5 in the **Online Supplementary Document**). However, the coefficients varied across two dimensions, with the least equitable healthcare resources by geographic dimension (**Table 2**). In 2021, the Gini coefficients by population dimension ranged from 0.081 to 0.185 for healthcare infrastructure resources, 0.053 to 0.082 for human resources, 0.116 to 0.162 for service resources, showing a high level of equality. On the contrary, the Gini coefficients by geographic dimension ranged from 0.578 to 0.651, 0.668 to 0.679, and 0.666 to 0.734 for corresponding three types of healthcare resource, close to or greater than 0.6, indicating a substantially higher level of inequity.

**Table 2:** Gini coefficients of healthcare resources in different dimensions in mainland China from 2010 to 2021

	Year											
**Resources**	**2010**	**2011**	**2012**	**2013**	**2014**	**2015**	**2016**	**2017**	**2018**	**2019**	**2020**	**2021**
Population dimension – healthcare infrastructure resources												
*Healthcare institutions*	0.188	0.190	0.191	0.192	0.194	0.193	0.194	0.196	0.194	0.189	0.184	0.179
*Primary healthcare institutions*	0.193	0.195	0.196	0.198	0.200	0.200	0.201	0.201	0.200	0.195	0.189	0.185
*Beds*	0.079	0.077	0.076	0.074	0.076	0.077	0.080	0.082	0.085	0.086	0.080	0.081
Equipment with a value of ≥CNY 10 000*	0.436	0.421	0.415	0.419	0.322	0.151	0.138	0.122	0.117	0.112	0.115	0.117
Population dimension – healthcare human resources												
*Healthcare technicians*	0.095	0.090	0.086	0.080	0.075	0.072	0.071	0.067	0.064	0.063	0.057	0.054
*Practising (assistant) physicians*	0.099	0.094	0.090	0.085	0.081	0.080	0.079	0.079	0.081	0.079	0.072	0.071
*Practising physicians*	0.121	0.118	0.116	0.112	0.107	0.104	0.100	0.098	0.097	0.092	0.085	0.082
*Registered nurses*	0.294	0.102	0.094	0.091	0.083	0.080	0.076	0.070	0.066	0.067	0.060	0.053
Population dimension – healthcare service resources												
*Total length of stay*	0.080	0.083	0.092	0.093	0.090	0.095	0.096	0.102	0.098	0.109	0.121	0.116
*Outpatient visits in healthcare institutions*	0.140	0.146	0.145	0.143	0.145	0.146	0.142	0.140	0.144	0.144	0.130	0.136
*Outpatient visits in primary healthcare institutions*	0.122	0.132	0.132	0.130	0.134	0.136	0.134	0.132	0.142	0.144	0.151	0.162
Geographic dimension – healthcare infrastructure resources												
*Healthcare institutions*	0.630	0.626	0.622	0.623	0.622	0.623	0.623	0.624	0.626	0.628	0.630	0.634
*Primary healthcare institutions*	0.632	0.627	0.623	0.625	0.625	0.625	0.625	0.626	0.628	0.630	0.632	0.636
*Number of beds*	0.648	0.647	0.648	0.647	0.646	0.647	0.649	0.647	0.646	0.646	0.650	0.651
Number of equipment with a value of ≥CNY 10 000*	0.799	0.796	0.791	0.788	0.723	0.612	0.607	0.586	0.574	0.570	0.579	0.578
Geographic dimension – healthcare human resources												
*Healthcare technicians*	0.666	0.665	0.667	0.667	0.666	0.667	0.667	0.666	0.668	0.667	0.668	0.668
*Practising (assistant) physicians*	0.661	0.662	0.666	0.668	0.669	0.671	0.672	0.672	0.676	0.675	0.677	0.677
*Practising physicians*	0.662	0.664	0.667	0.669	0.671	0.672	0.673	0.674	0.677	0.677	0.678	0.679
*Registered nurses*	0.731	0.675	0.676	0.677	0.675	0.675	0.674	0.671	0.672	0.670	0.669	0.668
Geographic dimension – healthcare service resources												
*Total length of stay*	0.650	0.651	0.650	0.650	0.654	0.656	0.656	0.656	0.659	0.656	0.665	0.666
*Outpatient visits in healthcare institutions*	0.708	0.712	0.714	0.715	0.717	0.718	0.717	0.716	0.718	0.718	0.715	0.719
*Outpatient visits in primary healthcare institutions*	0.701	0.706	0.708	0.710	0.713	0.715	0.715	0.716	0.722	0.723	0.724	0.734

*Including 15 provinces across eastern (Beijing, Liaoning, Jiangsu, Fujian, Shandong, and Guangdong)), central (Heilongjiang, Jiangxi, Hubei, and Hunan), and western regions (Inner Mongolia, Sichuan, Yunnan, Shaanxi, and Gansu) in mainland China.

### Theil index of healthcare resources

The total and intra-/inter-regional Theil index for almost all healthcare resource indicators continued to decline between 2010–21, indicating a narrowed inequity (**Table 3**). The index by geographic dimension was extremely greater than that by population dimension, indicating greater equity in healthcare resource allocation according to population dimension. In 2021, the intraregional Theil index for almost all indicators, except for the number of beds, was greater than the inter-regional Theil index in the population dimension was more than 85.95% for healthcare infrastructure resources, 76.47% for human resources, and 51.76% for service resources. Conversely, the inter-regional contribution rates were more than 60.81%, 62.75%, and 55.50% for three types of healthcare resources in the geographic dimension, respectively (**Table 3**).

**Table 3:** Theil index of healthcare resources in different dimensions in mainland China from 2010 to 2021*

	Year											
**Dimensions and resources**	**2010**	**2011**	**2012**	**2013**	**2014**	**2015**	**2016**	**2017**	**2018**	**2019**	**2020**	**2021**
**Population dimension – healthcare infrastructure**												
Healthcare institutions												
*T*	0.0610	0.0635	0.0639	0.0654	0.0659	0.0656	0.0658	0.0657	0.0643	0.0598	0.0562	0.0534
*T-intra*	0.0537	0.0544	0.0541	0.0549	0.0551	0.0548	0.0551	0.0559	0.0561	0.0524	0.0493	0.0475
*T-inter*	0.0074	0.0091	0.0098	0.0105	0.0108	0.0108	0.0106	0.0098	0.0083	0.0074	0.0068	0.0058
Primary healthcare institutions												
*T*	0.0649	0.0678	0.0683	0.0697	0.0705	0.0707	0.0704	0.0701	0.0685	0.0637	0.0600	0.0570
*T-intra*	0.0574	0.0584	0.0582	0.0596	0.0602	0.0602	0.0599	0.0604	0.0604	0.0565	0.0533	0.0513
*T-inter*	0.0076	0.0094	0.0100	0.0100	0.0103	0.0105	0.0106	0.0097	0.0080	0.0072	0.0067	0.0057
Beds												
*T*	0.0101	0.0094	0.0092	0.0090	0.0095	0.0096	0.0103	0.0111	0.0121	0.0122	0.0113	0.0115
*T-intra*	0.0080	0.0074	0.0072	0.0065	0.0062	0.0061	0.0063	0.0066	0.0066	0.0062	0.0053	0.0050
*T-inter*	0.0021	0.0020	0.0020	0.0025	0.0033	0.0035	0.0040	0.0046	0.0055	0.0060	0.0060	0.0065
Equipment with a value of ≥CNY 10 000†												
*T*	0.0693	0.0630	0.0567	0.0534	0.0467	0.0450	0.0469	0.0407	0.0374	0.0353	0.0348	0.0342
*T-intra*	0.0411	0.0383	0.0386	0.0363	0.0331	0.0347	0.0386	0.0330	0.0300	0.0284	0.0298	0.0294
*T-inter*	0.0282	0.0247	0.0181	0.0171	0.0136	0.0103	0.0084	0.0077	0.0074	0.0069	0.0051	0.0048
**Population dimension – healthcare human resources**												
Healthcare technicians												
*T*	0.0159	0.0145	0.0132	0.0111	0.0097	0.0092	0.0089	0.0082	0.0076	0.0074	0.0062	0.0057
*T-intra*	0.0125	0.0118	0.0109	0.0093	0.0085	0.0083	0.0080	0.0073	0.0066	0.0066	0.0056	0.0051
*T-inter*	0.0033	0.0027	0.0023	0.0017	0.0012	0.0009	0.0009	0.0009	0.0010	0.0008	0.0006	0.0006
Practising (assistant) physicians												
*T*	0.0162	0.0152	0.0137	0.0121	0.0111	0.0110	0.0108	0.0107	0.0112	0.0105	0.0088	0.0086
*T-intra*	0.0129	0.0122	0.0107	0.0095	0.0091	0.0091	0.0088	0.0087	0.0087	0.0083	0.0071	0.0072
*T-inter*	0.0034	0.0030	0.0029	0.0025	0.0019	0.0019	0.0020	0.0020	0.0025	0.0022	0.0016	0.0013
Practising physicians												
*T*	0.0238	0.0230	0.0221	0.0204	0.0187	0.0179	0.0168	0.0160	0.0158	0.0145	0.0127	0.0119
*T-intra*	0.0169	0.0164	0.0156	0.0144	0.0138	0.0134	0.0125	0.0119	0.0114	0.0105	0.0094	0.0091
*T-inter*	0.0069	0.0066	0.0065	0.0060	0.0048	0.0045	0.0043	0.0041	0.0044	0.0040	0.0033	0.0028
Registered nurses												
*T*	0.2042	0.0192	0.0170	0.0149	0.0125	0.0116	0.0105	0.0090	0.0080	0.0082	0.0068	0.0056
*T-intra*	0.1571	0.0155	0.0145	0.0129	0.0114	0.0109	0.0099	0.0085	0.0074	0.0079	0.0064	0.0052
*T-inter*	0.0471	0.0036	0.0025	0.0020	0.0011	0.0007	0.0006	0.0006	0.0007	0.0004	0.0004	0.0004
**Population dimension – healthcare service resources**												
Total length of stay												
*T*	0.0104	0.0112	0.0139	0.2288	0.2232	0.2232	0.0148	0.0171	0.0159	0.0188	0.0231	0.0212
*T-intra*	0.0089	0.0094	0.0101	0.1895	0.1851	0.1850	0.0101	0.0111	0.0100	0.0111	0.0127	0.0132
*T-inter*	0.0015	0.0018	0.0038	0.0393	0.0381	0.0382	0.0047	0.0060	0.0059	0.0077	0.0104	0.0081
Outpatient visits in healthcare institutions												
*T*	0.0246	0.0290	0.0289	0.0322	0.0332	0.0341	0.0330	0.0321	0.0340	0.0343	0.0299	0.0328
*T-intra*	0.0116	0.0158	0.0168	0.0128	0.0136	0.0144	0.0147	0.0142	0.0155	0.0166	0.0155	0.0173
*T-inter*	0.0130	0.0132	0.0121	0.0195	0.0196	0.0197	0.0183	0.0179	0.0185	0.0177	0.0144	0.0155
Outpatient visits in primary healthcare institutions												
*T*	0.0314	0.0341	0.0334	0.0282	0.0297	0.0313	0.0305	0.0301	0.0358	0.0385	0.0429	0.0512
*T-intra*	0.0121	0.0144	0.0145	0.0144	0.0151	0.0154	0.0155	0.0147	0.0177	0.0194	0.0233	0.0265
*T-inter*	0.0193	0.0197	0.0189	0.0137	0.0146	0.0158	0.0150	0.0154	0.0181	0.0191	0.0196	0.0247
**Geographic dimension – healthcare infrastructure resources**												
Healthcare institutions												
*T*	0.7081	0.6967	0.6868	0.6905	0.6889	0.6902	0.6904	0.6920	0.6981	0.7026	0.7093	0.7202
*T-intra*	0.2707	0.2642	0.2637	0.2678	0.2672	0.2662	0.2648	0.2645	0.2647	0.2637	0.2620	0.2608
*T-inter*	0.4374	0.4325	0.4231	0.4227	0.4217	0.4240	0.4257	0.4275	0.4334	0.4389	0.4473	0.4593
Primary healthcare institutions												
*T*	0.7138	0.7017	0.6916	0.6953	0.6956	0.6965	0.6960	0.6974	0.7037	0.7081	0.7139	0.7246
*T-intra*	0.2751	0.2682	0.2676	0.2681	0.2682	0.2687	0.2677	0.2672	0.2676	0.2665	0.2644	0.2630
*T-inter*	0.4388	0.4335	0.4241	0.4272	0.4274	0.4278	0.4284	0.4303	0.4361	0.4415	0.4495	0.4615
Beds												
*T*	0.7815	0.7768	0.7751	0.7709	0.7673	0.7687	0.7725	0.7666	0.7637	0.7620	0.7736	0.7765
*T-intra*	0.2776	0.2783	0.2843	0.2891	0.2902	0.2910	0.2962	0.2961	0.2960	0.2995	0.3020	0.3043
*T-inter*	0.5039	0.4984	0.4908	0.4818	0.4771	0.4777	0.4763	0.4705	0.4676	0.4626	0.4716	0.4722
Equipment with a value of ≥CNY 10 000†												
*T*	0.7972	0.7875	0.7562	0.7663	0.7446	0.6942	0.6926	0.6737	0.6436	0.6340	0.6478	0.6499
*T-intra*	0.2581	0.2558	0.2505	0.2535	0.2423	0.2511	0.2646	0.2434	0.2326	0.2257	0.2175	0.1878
*T-inter*	0.5392	0.5317	0.5057	0.5129	0.5023	0.4431	0.4280	0.4303	0.4111	0.4082	0.4303	0.4621
**Geographic dimension – healthcare human resources**												
Healthcare technicians												
*T*	0.8406	0.8393	0.8448	0.8425	0.8397	0.8403	0.8396	0.8379	0.8417	0.8380	0.8394	0.8401
*T-intra*	0.2712	0.2745	0.2815	0.2857	0.2885	0.2904	0.2938	0.2966	0.2979	0.2994	0.3023	0.3015
*T-inter*	0.5694	0.5649	0.5633	0.5568	0.5513	0.5499	0.5458	0.5413	0.5438	0.5386	0.5372	0.5385
Practising (assistant) physicians												
*T*	0.8216	0.8273	0.8376	0.8404	0.8449	0.8487	0.8518	0.8520	0.8605	0.8585	0.8622	0.8640
*T-intra*	0.2619	0.2666	0.2682	0.2677	0.2680	0.2663	0.2688	0.2684	0.2696	0.2718	0.2764	0.2799
*T-inter*	0.5597	0.5607	0.5694	0.5727	0.5769	0.5824	0.5830	0.5836	0.5910	0.5867	0.5858	0.5841
Practising physicians												
*T*	0.8362	0.8429	0.8524	0.8559	0.8611	0.8639	0.8661	0.8672	0.8750	0.8737	0.8772	0.8786
*T-intra*	0.2672	0.2719	0.2734	0.2730	0.2748	0.2752	0.2768	0.2767	0.2781	0.2813	0.2842	0.2872
*T-inter*	0.5691	0.5710	0.5789	0.5830	0.5863	0.5887	0.5892	0.5905	0.5970	0.5924	0.5930	0.5914
Registered nurses												
*T*	1.1093	0.8779	0.8792	0.8786	0.8696	0.8674	0.8622	0.8541	0.8557	0.8472	0.8440	0.8430
*T-intra*	0.2811	0.2928	0.2994	0.3006	0.3040	0.3067	0.3096	0.3131	0.3145	0.3155	0.3166	0.3140
*T-inter*	0.8282	0.5851	0.5798	0.5780	0.5656	0.5607	0.5527	0.5410	0.5412	0.5317	0.5275	0.5290
**Geographic dimension – healthcare service resources**												
Total length of stay												
*T*	0.7929	0.7868	0.7808	0.7795	0.7916	0.7934	0.7923	0.7925	0.8016	0.7985	0.8231	0.8299
*T-intra*	0.3190	0.3086	0.3206	0.3248	0.3228	0.3219	0.3189	0.3315	0.3334	0.3448	0.3659	0.3693
*T-inter*	0.4739	0.4783	0.4602	0.4547	0.4689	0.4715	0.4735	0.4610	0.4682	0.4537	0.4572	0.4607
Outpatient visits in healthcare institutions												
*T*	0.9921	1.0068	1.0092	1.0129	1.0235	1.0279	1.0236	1.0215	1.0303	1.0292	1.0073	1.0291
*T-intra*	0.3236	0.3237	0.3228	0.3198	0.3186	0.3229	0.3235	0.3250	0.3329	0.3374	0.3331	0.3424
*T-inter*	0.6684	0.6831	0.6863	0.6930	0.7048	0.7050	0.7000	0.6965	0.6974	0.6919	0.6742	0.6866
Outpatient visits in primary healthcare institutions												
*T*	0.9411	0.9552	0.9567	0.9640	0.9774	0.9850	0.9860	0.9929	1.0152	1.0200	1.0160	1.0494
*T-intra*	0.3188	0.3194	0.3149	0.3081	0.3015	0.3025	0.3027	0.3053	0.3168	0.3214	0.3254	0.3332
*T-inter*	0.6224	0.6358	0.6418	0.6559	0.6759	0.6825	0.6833	0.6876	0.6984	0.6986	0.6906	0.7162

T – Theil index, T-inter – inter-regional Theil index, T-intra – intra-regional Theil index

*The Theil index is a measure of economic inequality that allows for decomposition into inter-regional and intra-regional components. Inter-regional Theil index measures the inequality between different regions, and the intra-regional Theil index measures the inequality within each region.

†Including 15 provinces across eastern (Beijing, Liaoning, Jiangsu, Fujian, Shandong, and Guangdong), central (Heilongjiang, Jiangxi, Hubei, and Hunan), and western regions (Inner Mongolia, Sichuan, Yunnan, Shaanxi, and Gansu) in mainland China.

## DISCUSSION

In this descriptive study, we used panel data from 31 provinces in mainland China. There was a substantial increase in the densities of healthcare resources, such as healthcare institutions and technicians, from 2010 to 2021, alongside a significant improvement in healthcare services. A wide variation is exhibited in healthcare resources across geographic regions and provinces. Gini coefficients showed a higher inequity in resource allocation by geographic dimension (>0.6) compared to population (<0.2). The intraregional Theil index based on population surpassed the inter-regional index, with contribution rates exceeding 60%, while variation differs by geographic dimension.

Previous evidence showed a profound transformation in China’s healthcare landscape over the past decades, presenting an effective reform within the sector [9,28]. A nationwide longitudinal study in mainland China from 2012 to 2021, despite using a different indicator, showed a significant upward trajectory in the number of general physicians per 10 000 population, increasing from 0.81 in 2012 to 3.08 per 10 000 population in 2021 [15], reflecting a substantial improvement in healthcare personnel density. Similar findings were observed in several studies conducted in China, including Guangdong between 2017–21 [16] and Western China between 2014–18, which focused on physicians and nurses [17]. Furthermore, several population-based cohort studies in China consistently demonstrated a decline in length of stay, with pneumonia hospitalisations showing a –1.0% annualised change from 2009 to 2017 [18], and avoidable hospitalisations due to chronic diseases (*e.g.* hypertension, diabetes, asthma) exhibiting a range between –5.2% and –10.5% from 2015 to 2018 [19]. In our study, the notable improvement in healthcare resource allocation, such as increase in equipment valued at ≥CNY 10 000 and healthcare technicians were observed, which might be driven by various factors, including policy initiatives, technological advancements, and rising demand for healthcare services. In the past decades, China has introduced several policy initiatives, such as the Universal Health Coverage, Health China 2030 Plan, to facilitate a large shift in healthcare infrastructure expansion and personnel augmentation [10,13,29]. In 2009, China initiated policies to encourage private investments in the healthcare industry to expand the healthcare supply [30], significantly increasing the number of private hospitals to 66% of the total by 2020 and healthcare technicians [31]. Health insurance management agencies established performance evaluation indicators, such as turnover efficiency, to enhance the healthcare service capacity for public hospitals, consequently reducing the length of stay for every patient [28]. In addition, the advent of transformative technologies within medicine, including telemedicine, wearable devices enabling remote monitoring, and artificial intelligence-driven diagnosis and treatment, optimised patient care pathways, stimulating the acquisition and utilisation of advanced medical equipment while increasing the demand for skilled healthcare professionals, stimulating the acquisition and utilisation of advanced medical equipment while increasing the demand for skilled healthcare professional [32,33]. Moreover, the rising demand for healthcare services, driven by an ageing population and a growing prevalence of chronic diseases, has expanded the number of advanced medical equipment and healthcare professionals.

Despite the achievements, our study also highlighted substantial geographic variation in healthcare resources within China. For example, in 2021, the density of equipment valued at ≥CNY 10 000 was 8.13 per 1000 population in the eastern region, higher than 6.72 per 1000 population in the central region, about five times higher in the province with the highest estimate (22.82 per 1000 population in Beijing) than that with the lowest estimate (4.93 per 1000 population in Jiangxi). In addition, three of five hotspot clusters of higher density of practising (assistant) physicians were observed in eastern regions, and two of four cold-spot clusters of lower density were concentrated in the western regions. To assess this further, we examined the healthcare resources across GDP tertiles and urbanisation tertiles to evaluate the effect of economic factors on resource distribution. We found that healthcare resources were more abundant in regions with higher GDP and urbanisation levels than those with lower levels (Table S6 in the **Online Supplementary Document**). Digital economy development, associated with regional economy and urbanisation [34], might increase infrastructure investment, leading to higher densities of high-value equipment and personnel in developed regions like the eastern provinces compared to those less developed regions in central and western regions [35,36]. Additionally, with the economic and social system reforms, various healthcare resources have gradually converged towards the developed eastern regions and metropolis, accentuating the disparity in healthcare resource distribution between eastern and central-western regions [9]. For example, regions targeted for increased investment might expand their healthcare infrastructure and attract skilled professionals, enhancing service quality and access; regions with limited financial support might experience insufficient facilities and healthcare personnel, exacerbating disparities in access to care. Despite efforts like the Western Development Strategy and the Plan on Revitalizing Northeast, which have allocated substantial central fiscal transfers to the underdeveloped western regions and leaded to a continuous growth in healthcare investments [37], significant regional imbalances persist. This is partly due to the slow pace of infrastructure development in western regions and ongoing economic disparities. Additionally, existing policies may lack comprehensive strategies to effectively address the natural causes of inequities, such as socioeconomic factors and historical resource allocation patterns. To further enhance healthcare resource availability and accessibility in the western regions, the government could consider strategies such as establishing hierarchical diagnosis and treatment systems, medical treatment alliances, and increasing financial investment.

We showed that the Gini coefficient by population for healthcare resources was <0.2, indicating relatively equitable allocation, suggesting an improvement in the average access to healthcare services among residents. However, the Gini coefficient by geographic dimension exceeds 0.6, indicating high inequity. China’s healthcare system predominantly adopts a supply-driven approach in resource allocation, primarily focusing on the available number of health resources per 1000 population while neglecting the impact of geographic factors and spatial distribution on health resource allocation [38]. This results in a disparity where healthcare resources were mainly concentrated in densely populated and developed municipalities and eastern regions, leaving the vast and sparsely populated western regions severely underserved. Moreover, regional health plans implemented by provincial governments in recent years might facilitate the equitable development in healthcare resource allocation [39]. The findings from Theil index indicate that the inequity in healthcare resource allocation is predominantly caused by intraregional disparities. Although national policies aimed at narrowing the gap in healthcare resource allocation between developed and underdeveloped regions have yielded some achievements in ameliorating inter-regional inequities, national policies and strategies should also address intraregional disparities in health resource allocation. In addition, further studies are essential to assess the implications of our findings for the implementation of national policies.

Our study has several limitations, the most important being the use of aggregated data, so we cannot evaluate the distribution and equity of healthcare resources accounting for several confounders, including political factors, actual health status, and health service demand. The health resource data for selected provinces have not yet been validated via on-site visits, and health resource density is likely underestimated, as institutions in areas with limited resources and underdeveloped information systems are more likely to underreport data. In addition, the number of equipment valued at ≥CNY 10 000 at the provincial level was not reported by the National Health Commission of China. Therefore, we acquired data from all provincial health commissions and received responses from 15 provinces covering mainland China's eastern, central, and western regions. Although the potential for selection bias, the national density of equipment valued at ≥CNY 10 000 per 1000 population was found to be slightly lower than the figure in 2010 (2.11 per 1000 population) but close to the figure in 2021 (7.43 per 1000 population) for 31 provinces from China Health Statistics Yearbook. Because of ignoring the uneven distribution within the province, the Gini coefficient calculated in this study is much smaller than the actual Gini coefficient of medical resources in China calculated in each person. Despite these limitations, this study is the first to characterise geographic variations and temporal trends in the distribution and inequity of healthcare resources over a 12-year period in mainland China. Using census rather than sample data from the China Health Statistics Yearbook, an annual statistical reference book compiled by the National Health Commission of China, substantially enhances the reliability and credibility of our findings. To the best of our knowledge, our study is the most comprehensive analysis to date of healthcare resources encompassing infrastructure, human, and service resources, providing a detailed understanding of disparity and development in healthcare provision across mainland China.

## CONCLUSIONS

Between 2010–21, the per capita access to healthcare resources in China steadily increased, but significant geographic variations were observed, with hotspots of higher resource density concentrated in eastern regions and cold spots predominantly in western regions. The equity of healthcare resources allocation by population is superior to that by geographic dimension, and intra-regional variation based on population surpasses inter-regional variation. Investments in comprehensive and effective strategies to reduce disparities and enhance resource allocation are urgently required. Increased attention should be given to western regions as well as in other under-resourced areas settings. China’s experience in improving the allocation and equity of healthcare resources can serve as an example for other LMICs.

## Additional material


Online Supplementary Document
