# Supplementary material for: Geographic variations, temporal trends, and equity in healthcare resource allocation in China, 2010–21
Source: J Glob Health. 2025 Jan 17;15:04008. doi: 10.7189/jogh.15.04008 (PMC11737812; doi:10.7189/jogh.15.04008)
Supplement: Online Supplementary Document [file jogh-15-04008-s001.pdf]

Supplement to: Yin S, Liu Z, Yu S, Li Y, An Q, Wang D, Yan H, Xiao Y, Xu F, Tian Y, Luan X. Geographic variations, temporal trends, and equity in healthcare resource allocation in China, 2010–21. J Glob Health. 2025;15:04008.

Method S1

Global Moran's *I* Index

The Global Moran's I index, an exploratory spatial data analysis (ESDA), was conducted on the aggregation index of eight major health resource indicators across 31 provinces in China. The model formula is as follows:

$$\text{Global Moran's } I = \frac{n}{\sum_{i=1}^{n}\sum_{j=1}^{n} w_{ij}} \cdot \frac{\sum_{i=1}^{n}\sum_{j=1}^{n} w_{ij}(x_i-\bar{x})(x_j-\bar{x})}{\sum_{i=1}^{n}(x_i-\bar{x})^2} \quad i \neq j \qquad (1)$$

Where x_i and x_j represent the observed values of the health resource aggregation index in spatial province units, $\bar{x}$ is the aggregation index of health resources in the spatial region of interest. W_{ij} is the spatial weight matrix defined by the Rook adjacency criterion, and n is the total number of provinces. The Moran's I index typically ranges from (-1, 1). A value less than 0 indicates a high negative correlation in spatial aggregation of health resources between provinces; a value of 0 indicates no spatial correlation in the aggregation of health resources between provinces, while a value greater than 0 indicates a high positive correlation in the spatial aggregation of health resources between provinces. The Moran's *I* index can be tested for significance through the standardization of the *Z* statistic, calculated as follows:

$$Z = \frac{I-E(I)}{\sqrt{VAR(I)}} \qquad (2)$$

Where *E(I)* and *VAR(I)* denote the expected value and variance of *Moran's I*, respectively. A positive and significant Z value suggests spatial autocorrelation in health resource aggregation, indicating a tendency towards clustered distribution of similar health resource observations. A negative and significant Z value indicates spatial negative autocorrelation, where similar health resource observations tend to be dispersed. A Z value of 0 suggests independent random distribution of observations. We calculated the *Global Moran's I* and the *Z* value using the density of healthcare institutions as the example.

1. Define Variables

- Number of provinces (n): 31
- Density of healthcare institutions (xi): The density for each province is calculated as:

$$\text{Density} = \frac{\text{Number of Healthcare institutions}}{\text{Population}} \times 10000$$

- Mean density ($\bar{x}$)

$$\text{Density} = \frac{33051 + 10699 + \cdots + 6076}{42290000 + 21890000 + \cdots + 13730000} \times 10000 = 7.30$$

- Spatial Weight Matrix (W): Constructed based on the adjacency relationships between provinces*.

Province	Anhui	Beijing	Fujian	…	Xinjiang	Yunnan	Zhejiang	Chongqing
Anhui	0	0	0	…	0	0	1	0
Beijing	0	0	0	…	0	0	0	0
Fujian	0	0	0	…	0	0	1	0
Gansu	0	0	0	…	1	0	0	0
Guangdong	0	0	1	…	0	0	0	0
…	…	…	…	…	…	…	…	…
Guizhou	0	0	0	…	0	1	0	1
Yunnan	0	0	0	…	0	0	0	0
Zhejiang	1	0	1	…	0	0	0	0
Chongqing	0	0	0	…	0	0	0	0

*The rows and columns of the matrix correspond to each province. A value of 1 indicates adjacency, while a value of 0 indicates non-adjacency.

2. Calculate the Numerator

The numerator for the Global Moran's I is calculated as:

$$\text{Numerator}=\sum_{i=1}^{n}\sum_{j=1}^{n} w_{ij}(x_i - \bar{x})(x_j - \bar{x})=1.298$$

Where w_{ij}=1 if provinces i and j are neighbors, and 0 otherwise.

3. Calculate the Denominator

The denominator is computed as:

$$\text{Denominator}=\sum_{i=1}^{n}(x_i - \bar{x})^2 =6.50$$

4. Compute the Total Weight

Calculate the total number of neighboring connections:

$$S=\sum_{i=1}^{n}\sum_{j=1}^{n} w_{ij} (S = 135)$$

5. Calculate *Global Moran's I*

Using the values from above results:

$$\textbf{\textit{Global Moran's I}} = \frac{n}{S} \cdot \frac{Numerator}{\text{Denominator}} = 0.04586$$

6. Calculate the *Z* Value

$$\text{VAR}(I) = \frac{(n^2-1)(\sum wij) - 2\sum w^2 ij}{n^2(n-1)^2}$$

where n=31, $\sum wij$=135,

$$\text{VAR}(I) = \frac{(31^2-1)\times 135 - 2\times 135}{31^2(31-1)^2} = 0.149$$

$$Z = \frac{0.04586-(-0.40216)}{\sqrt{0.149}} = 1.16066$$

Local Moran's I Index

To further investigate the local interaction between provinces and surrounding areas, this study employed Local Moran's I index to measure the spatial distribution of health resource aggregation in China, as well as the degree of spatial differentiation between provincial and neighboring areas. The model formula is as follows:

$$\text{Local Moran's } I = \frac{\text{n}(x_i-\bar{x})\sum_j^n w_{ij}(x_j-\bar{x})}{\sum_{i=1}^n (x_i-\bar{x})^2} \qquad (3)$$

Where $(x_i - \bar{x})$ represents the deviation of the observed value from the mean on the i provincial unit. x_i, x_j, $\bar{x}$, w_{ij} and n have the same meanings as in above equation (1). Similarly, the standardized Moran's I can be obtained and tested for significance. At a given significance level (P< 0.05), if the local Moran's I>0, it indicates small spatial differentiation in health resource aggregation, with provincial health resource aggregation exhibiting similar spatial clustering phenomena to neighboring provinces. Conversely, if local Moran's I<0, it suggests large spatial differentiation in health resource aggregation, indicating a distribution of health resources in neighboring provinces that differs from the focal province. Additionally, for visualization purposes, the local Moran's I analysis can be used to generate a LISA (Local Indicators of Spatial Association) distribution map, which categorizes results into four significant types: High-High (HH), High-Low (HL), Low-Low (LL), and Low-High (LH), as well as one non-significant type, No Group (NG). These types represent different

spatial heterogeneity characteristics among local data. Specifically, HH and LL types indicate clustered distribution, where the level of a resource in a province is higher/lower than the average and neighboring provinces also have higher/lower levels than the average, showing local positive correlation. HL and LH types represent a pattern of alternating high and low, exhibiting a uniform distribution where the level of a resource in a province is higher/lower than the average while neighboring provinces have lower/higher levels than the average, indicating local negative correlation.

The local Moran's I was tested by calculating Z scores and their p-values:

1. Calculation of Z-score: The Z-score is obtained by normalizing the local Moran's I values, using the formula:

$$Z_i = \frac{I_i - E(I_i)}{SD(I_i)}$$

where $E(Ii)$ is the expected value of the local Moran's I and $SD(Ii)$ is its standard deviation.

2.Monte Carlo simulation was used to examine the significance:

(1) Calculate the original local Moran's I: Compute the local Moran's I value for each province using the provided actual data, resulting in a list of original local Moran's I values.

(2) Prepare for Monte Carlo simulation: Determine the number of simulations, typically choosing 1000 or more for stable results.

Randomly permute the observed values: Randomly shuffle the observed values. For example, if you have density values for healthcare technicians, you can use random functions in programming languages (like Python or R) to shuffle these values.

(3) Recalculate local Moran's I: For each simulation, recalculate the local Moran's I values for each province using the permuted data. This step generates 1000 local Moran's I values.

(4) Construct the empirical distribution: Aggregate all 1000 simulated local Moran's I values to construct their distribution.

(5) Compare the original value with the simulated distribution: Determine the position of the original local Moran's I value within the simulated distribution. You

can calculate the p-value by assessing the frequency of the original value being greater than or less than the simulated values.

(6) Calculating how many simulated local Moran's I values are greater than the original value and using the following formula to compute the p-value:

$$p - value = \frac{\text{number of simulated I values} \geq \text{original I}}{\text{Total number of simulations}}$$

Lorenz Curve

The Lorenz curve is a graphical representation of the distribution of resources among a population. It plots the cumulative percentage of healthcare resources on the Y-axis against the cumulative percentage of the population (or geographical area) ranked in ascending order on the X-axis. The 45-degree diagonal line represents perfect equality, where each percentage of the population receives an equal share of resources. Deviations from this line indicate varying degrees of inequality; the greater the curvature of the Lorenz curve away from the line of equality, the higher the level of inequity present in the distribution of resources.

The Lorenz curve can be mathematically expressed by plotting the cumulative share of the population, L(p), where p is the cumulative proportion of the population, against the cumulative share of resources, $R(p)$:

$$L(p) = \frac{\sum_{i=1}^{n} y_i}{\sum_{i=1}^{n} y_i \cdot n}$$

where y_i is the resource allocation for the i-th region, and n is the total number of regions.

Gini Coefficient

The Gini coefficient quantifies the degree of inequality represented by the Lorenz curve. It is calculated as the ratio of the area between the Lorenz curve and the line of perfect equality to the total area under the line of perfect equality. The Gini coefficient G can be expressed mathematically as:

$$G = \frac{A}{A + B}$$

where A is the area between the Lorenz curve and the line of perfect equality, and B is the area under the Lorenz curve. Alternatively, it can be calculated using the formula:

$$G = 1\text{-}2\int_0^1 L(p)dp$$

The Gini coefficient ranges from 0 to 1, where 0 indicates perfect equality and 1 indicates perfect inequality. To interpret the Gini coefficient, thresholds are established: values below 0.2 indicate perfect equality, 0.2 to 0.3 signify relative equality, 0.3 to 0.4 reflect moderate inequality, 0.4 to 0.5 suggest relative inequity, and values above 0.5 indicate severe inequity in resource distribution.

Theil Index

The Theil index is a measure of economic inequality that allows for decomposition into inter-regional and intra-regional components, providing insights into the sources of inequity. It is calculated based on the proportion of each province's population or geographical area relative to the total population or area, as well as the distribution of healthcare resources within each province. The Theil index T can be expressed as:

$$T = \sum_{i=1}^{n} \frac{yi}{\bar{y}} \ln(\frac{yi}{y})$$

where yi is the resource allocation for the i-th region, and $\bar{y}$ is the mean resource allocation across all regions. The Theil index starts at 0, indicating no inequality, and increases indefinitely, allowing for a comprehensive assessment of disparities. Its ability to decompose into inter-regional and intra-regional components makes it particularly useful for identifying specific areas of concern in healthcare resource allocation.

Table S1. Definitions of indicators of healthcare resources

Variable	Definition
Number of healthcare institutions	At the end of the year, the sum number of healthcare institutions encompass hospitals, primary healthcare facilities, specialized public health institutions, and other healthcare facilities.
Number of beds	At the end of the year, the total fixed actual beds (excluding authorized beds) in healthcare institutions, including regular beds, makeshift beds, intensive care unit (ICU) beds, beds undergoing disinfection and repair, and beds temporarily out of service due to expansion or major renovations.
Number of equipment with a value of ⩾CNY 10 000	At the end of the year, the amount of equipment with a unit price exceeding CNY 10 000 in healthcare institutions.
Number of healthcare technicians	At the end of the year, the sum of healthcare technicians includes practicing physicians, practicing (assistant) physicians, registered nurses, pharmacists, laboratory technicians, radiology technicians, health inspectors, and interns in medical, pharmaceutical, nursing, and technical fields. Excluded from this category are healthcare technicians engaged in managerial roles such as hospital directors, vice directors, and party secretaries.
Number of practicing (assistant) physicians	At the end of the year, the sum of physicians who have hold a "Practicing Assistant Physician" qualification in their physician practicing certificates and actively engaged in medical and preventive healthcare services, excluding those involved in managerial roles.
Number of practicing physicians	At the end of the year, the sum of physicians holding a "Practicing Physician" qualification in their physician practicing certificates and actively engaged in medical and preventive healthcare services, excluding those involved in managerial roles.
Number of registered nurses	At the end of the year, the sum of nurses who have obtained the certificate of registered nurses and are actually engaged in nursing work in healthcare institutions.
Beds occupancy rate	The ratio calculated as the total actual occupied bed days divided by the total actual available bed days, multiplied by 100%.

Average length of stay	The ratio calculated as the total bed days occupied by discharged patients divided by the number of discharged patients.
Number of outpatient visits in healthcare institutions	At the end of the year, the sum of patient visits for medical consultations, which are counted based on registration numbers. This includes visits to outpatient departments, emergency rooms, off-site consultations, scheduled appointments, individual health check-ups, and health consultation sessions (excluding health lectures). If a patient registers for multiple visits during a single appointment, each visit is counted separately. However, procedures such as tests, treatments, and interventions ordered by medical professionals, as well as immunizations and health management services, are not included in this count. Additionally, visits by patients who do not register, visits by staff within the institution, and off-site consultations (excluding consultations conducted off-site) where registration fees are not charged are also counted as individual patient visits.

Table S2. The distribution of healthcare resources across geographic region in mainland China, 2010-2021

	Geographic regions	2010	2015	2021
**Health infrastructure resources**				
Number of healthcare institutions/10 000 population	Eastern	6.17	6.07	6.49
	Central	7.31	7.47	7.72
	Western	8.01	8.38	8.16
Number of primary healthcare institutions/10 000 population	Eastern	5.93	5.70	6.14
	Central	7.05	7.00	7.35
	Western	7.71	7.81	7.73
Number of equipment with a value of ⩾CNY 10 000 /1000 population*	Eastern	2.78	4.61	8.13
	Central	1.25	2.27	6.72
	Western	1.98	3.83	7.16
**Health human resources**				
Number of healthcare technicians/1000 population	Eastern	4.74	6.01	8.06
	Central	4.24	5.53	7.64
	Western	4.07	5.75	8.16
Number of practicing (assistant) physicians/1000 population	Eastern	1.93	2.33	3.19
	Central	1.76	2.15	2.96
	Western	1.69	2.05	2.88
Number of practicing physicians/1000 population	Eastern	1.63	1.98	2.73
	Central	1.39	1.71	2.42
	Western	1.36	1.68	2.39
Number of registered nurses/1000 population	Eastern	1.71	2.45	3.55
	Central	1.45	2.25	3.46
	Western	3.03	2.29	3.68
**Health services resources**				

Beds occupancy rate, %	Eastern	87.60	85.50	74.20
	Central	84.80	85.70	73.60
	Western	87.50	84.70	76.20
Number of beds/1000 population	Eastern	3.59	4.71	5.93
	Central	3.56	5.28	7.32
	Western	3.62	5.43	7.24
Average length of stay	Eastern	10.70	9.60	9.10
	Central	10.40	9.80	9.50
	Western	10.40	9.30	9.20
Number of outpatient visits in healthcare institutions	Eastern	2907655205	3933930850	4215730696
	Central	1520829333	1975161323	2192129000
	Western	1409101304	1784332956	2064173740
Number of outpatient visits in primary healthcare institutions	Eastern	166340	208977	206349
	Central	102086	122102	118226
	Western	92730	103115	100448

The total distribution of healthcare resources was summarized using absolute number or ratio for continuous variables, as appropriate.

*Including 15 provinces across eastern (Beijing, Liaoning, Jiangsu, Fujian, Shandong, and Guangdong)), central (Heilongjiang, Jiangxi, Hubei, and Hunan), and western regions (Inner Mongolia, Sichuan, Yunnan, Shaanxi, and Gansu) in mainland China.

Table S3. The distribution of healthcare resources across geographic region in mainland China in 2021, accounting for provincial socioeconomic factors*

Province	Number of healthcare institutions/ 10 000 population	Number of primary healthcare institutions/10 000 population	Number of equipment with a value of ≥CNY 10 000 /1000 population#	Number of healthcare technicians/1000 population	Number of practicing (assistant) physicians/1000 population	Number of practicing physicians/1000 population	Number of registered nurses/1000 population	Beds occupancy rate, %	Average length of stay, days	Number of beds/1000 population
Anhui	8.22	7.82		7.72	2.94	8.22	3.47	72.67	9.08	6.89
Beijing	3.94	3.58	16.73	9.70	3.76	3.94	4.22	74.90	9.36	6.03
Chongqing	7.61	7.14		8.78	3.38	7.61	3.79	71.06	9.43	6.88
Fujian	5.59	5.27	10.10	8.20	3.09	5.59	3.67	76.34	8.63	5.93
Gansu	10.07	9.57	4.94	7.71	2.90	10.07	3.41	68.31	9.11	7.32
Guangdong	3.82	3.62	9.68	7.83	2.94	3.82	3.51	84.07	8.77	5.09
Guangxi	9.17	8.70		7.61	2.76	9.17	3.27	72.29	8.46	6.33
Guizhou	9.80	9.30		7.66	2.80	9.80	3.26	71.55	8.50	6.61
Hainan	7.19	6.78		7.85	2.82	7.19	3.46	73.10	8.52	5.72
Hebei	8.48	8.07		7.91	3.09	8.48	3.55	72.59	9.15	7.36
Henan	9.21	8.79		7.46	2.85	9.21	3.27	74.57	8.92	7.01
Heilongjiang	9.41	8.86	9.12	8.80	3.46	9.41	3.86	65.31	10.42	7.98
Hubei	7.36	6.97	7.95	8.00	3.05	7.36	3.60	72.93	9.34	6.69
Hunan	8.36	7.94	6.10	7.76	2.93	8.36	3.44	72.91	9.08	6.75
Jilin	9.78	9.22		8.64	3.39	9.78	3.79	65.40	10.27	7.97
Jiangsu	4.84	4.61	7.45	7.98	3.19	4.84	3.70	80.37	9.61	6.55
Jiangxi	7.68	7.27	7.90	7.92	2.93	7.68	3.47	73.41	8.56	6.24
Liaoning	8.35	7.84	10.28	9.17	3.65	8.35	3.97	68.20	10.27	7.79
Inner	7.11	6.69	10.35	8.57	3.30	7.11	3.85	70.04	9.46	6.96

Mongolia										
Ningxia	7.32	6.89		8.46	3.17	7.32	3.70	71.65	8.53	6.45
Qinghai	9.73	9.18		8.41	3.15	9.73	3.52	69.33	8.87	6.97
Shandong	6.79	6.49	5.14	7.59	2.96	6.79	3.49	77.26	9.17	6.75
Shanxi	8.05	7.61		8.25	3.15	8.05	3.67	70.12	9.32	6.98
Shaanxi	7.82	7.40	8.09	8.16	3.11	7.82	3.63	71.53	9.16	6.84
Shanghai	3.28	2.92		9.88	3.80	3.28	4.34	73.53	9.42	5.81
Sichuan	9.65	9.18	4.50	7.77	3.02	9.65	3.44	70.56	9.84	7.54
Tianjin	4.26	3.88		9.68	3.71	4.26	4.21	73.13	9.06	5.97
Tibet	13.66	13.00		6.84	2.33	13.66	2.79	66.20	8.69	6.89
Xinjiang	8.60	8.15		7.78	2.85	8.60	3.40	71.62	8.50	6.39
Yunnan	10.32	9.82	4.26	7.44	2.75	10.32	3.27	69.29	9.18	7.08
Zhejiang	4.76	4.49		8.21	3.16	4.76	3.80	76.36	9.40	6.13

*The values were estimated by using generalized linear model accounting for gross domestic product (GDP), the proportion of women, urbanization rate, marriage rate, and the percentage of the population aged 15 and older.

#Including 15 provinces across eastern (Beijing, Liaoning, Jiangsu, Fujian, Shandong, and Guangdong)), central (Heilongjiang, Jiangxi, Hubei, and Hunan), and western regions (Inner Mongolia, Sichuan, Yunnan, Shaanxi, and Gansu) in mainland China.

Table S4. The distribution of healthcare resources across GDP tertiles and urbanization tertiles in mainland China in 2021

	Number of healthcare institutions/10 000 population		Number of primary healthcare institutions/10 000 population		Number of equipment with a value of ⩾CNY 10 000 /1000 population*		Number of healthcare technicians/1000 population		Number of practicing (assistant) physicians/1000 population	
Province	*Z* score	*P* value	*Z* score	*P* value	*Z* score	*P* value	*Z* score	*P* value	*Z* score	*P* value
Shanghai	2.507	**0.012**	2.499	**0.012**			0.483	0.629	0.513	0.608
Yunnan	1.324	0.185	1.375	0.169	0.304	0.761	0.146	0.884	1.793	0.073
Inner Mongolia	1.541	0.123	1.500	0.134			0.322	0.748	3.444	**0.001**
Beijing	-0.125	0.900	-0.136	0.892			0.091	0.927	6.698	**0.000**
Jilin	0.368	0.713	0.311	0.756			0.136	0.892	2.971	**0.003**
Sichuan	1.352	0.176	1.370	0.171	0.390	0.697	0.138	0.891	0.588	0.557
Tianjin	-0.277	0.782	-0.301	0.764			2.091	**0.037**	5.308	**0.003**
Ningxia	-0.547	0.584	-0.569	0.569			0.236	0.813	0.155	0.877
Anhui	0.968	0.333	0.906	0.365			0.753	0.452	-0.096	0.923
Shandong	-0.007	0.995	-0.008	0.994			-0.147	0.883	1.621	0.105
Shanxi	1.974	0.048	2.024	**0.043**			0.060	0.952	0.852	0.394
Guangdong	0.050	0.960	-0.013	0.990			1.781	0.075	3.016	**0.003**
Guangxi	0.346	0.730	0.310	0.757			0.458	0.647	2.924	**0.003**
Xinjiang	1.275	0.202	1.431	0.152			0.271	0.786	0.409	0.682
Jiangsu	2.071	**0.038**	2.057	**0.040**			0.064	0.949	0.317	0.751
Jiangxi	-0.100	0.920	-0.139	0.889			2.269	**0.023**	2.188	**0.029**
Hebei	0.302	0.702	0.369	0.712			-1.320	0.187	3.134	**0.002**
Henan	0.151	0.880	0.181	0.856			0.293	0.770	0.125	0.900
Zhejiang	1.620	0.105	1.569	0.117			-0.688	0.491	-0.764	0.445
Hainan	0.050	0.960	-0.013	0.990			1.781	0.075	1.267	0.205
Hubei	0.219	0.827	0.185	0.853	0.179	0.858	0.530	0.596	0.486	0.627

Hunan	-0.115	0.908	-0.131	0.895	0.172	0.864	1.016	0.310	1.250	0.211
Gansu	0.860	0.390	0.803	0.422	0.532	0.595	0.020	0.984	0.166	0.868
Fujian	0.375	0.707	0.323	0.747			1.410	0.159	1.671	0.095
Tibet	1.160	0.246	0.946	0.344			0.169	0.866	0.235	0.814
Guizhou	0.099	0.921	0.101	0.920			0.209	0.834	2.201	**0.028**
Liaoning	0.111	0.912	0.056	0.955	-0.155	0.877	-0.074	0.941	0.210	0.834
Chongqing	-0.028	0.978	-0.034	0.973	0.474	0.636	0.112	0.911	1.327	0.184
Shaanxi	0.466	0.641	0.477	0.634	0.172	0.864	-0.256	0.798	0.117	0.907
Qinghai	2.635	**0.008**	2.461	**0.014**			-0.391	0.696	-0.002	0.998
Heilongjiang	-0.472	0.64	-0.553	0.580	-0.028	0.978	-0.197	0.844	0.032	0.975

Table S4. The distribution of healthcare resources across GDP tertiles and urbanization tertiles in mainland China in 2021 (continuous)

	Number of practicing physicians/1000 population		Number of registered nurses/1000 population		Beds occupancy rate, %		Average length of stay, days	
Province	*Z* score	*P* value	*Z* score	*P* value	*Z* score	*P* value	*Z* score	*P* value
Shanghai	1.022	0.307	0.451	0.652	2.648	**0.008**	0.156	0.876
Yunnan	1.303	0.193	-0.700	0.484	0.464	0.642	0.650	0.516
Inner Mongolia	0.381	0.703	0.178	0.859	4.502	**0.000**	0.746	0.455
Beijing	5.749	**0.000**	-4.551	**0.000**	-0.021	0.983	0.358	0.720
Jilin	0.761	0.447	-0.016	0.987	2.573	**0.010**	2.889	**0.004**
Sichuan	0.523	0.601	0.051	0.960	-0.546	0.585	-3.141	**0.002**
Tianjin	0.568	**0.000**	-0.772	0.440	0.198	0.843	0.327	0.744
Ningxia	0.047	0.963	0.285	0.775	0.884	0.377	0.666	0.506
Anhui	0.312	0.755	0.309	0.757	-0.367	0.714	0.083	0.939
Shandong	-0.085	0.932	-0.144	0.886	0.256	0.798	-0.081	0.935
Shanxi	0.049	0.961	0.107	0.915	0.501	0.616	0.556	0.578
Guangdong	1.602	0.092	0.573	0.567	0.407	0.684	0.302	0.763

Guangxi	1.717	0.086	0.079	0.937	1.515	0.130	0.926	0.354
Xinjiang	0.641	0.522	1.094	0.274	-0.230	0.818	2.150	**0.032**
Jiangsu	0.209	0.834	0.108	0.914	1.184	0.236	0.054	0.957
Jiangxi	1.284	0.199	0.932	0.352	0.683	0.495	0.250	0.802
Hebei	0.223	0.823	-1.542	0.123	0.649	0.517	0.145	0.885
Henan	0.328	0.743	0.275	0.784	-0.008	0.994	0.245	0.806
Zhejiang	-0.516	0.606	-0.214	0.830	1.631	0.103	-0.002	0.999
Hainan	1.602	0.092	0.573	0.567	0.407	0.684	0.302	0.763
Hubei	0.725	0.469	0.008	0.994	1.122	0.262	0.197	0.844
Hunan	1.486	0.137	0.097	0.923	1.338	0.181	-0.191	0.848
Gansu	0.214	0.831	0.153	0.878	0.377	0.706	0.406	0.685
Fujian	0.541	0.588	0.911	0.362	0.215	0.829	0.616	0.538
Tibet	0.763	0.445	0.041	0.967	-1.552	0.121	0.355	0.723
Guizhou	1.443	0.149	0.132	0.895	1.300	0.194	-0.946	0.344
Liaoning	0.433	0.665	0.069	0.945	2.336	**0.019**	1.358	0.174
Chongqing	0.723	0.470	0.025	0.980	1.306	0.192	0.490	0.624
Shaanxi	0.289	0.773	0.169	0.866	0.109	0.913	-0.216	0.829
Qinghai	0.033	0.973	0.138	0.890	0.505	0.613	0.313	0.754
Heilongjiang	0.106	0.916	-0.255	0.799	4.034	**0.000**	2.671	0.008

*Including 15 provinces across eastern (Beijing, Liaoning, Jiangsu, Fujian, Shandong, and Guangdong)), central (Heilongjiang, Jiangxi, Hubei, and Hunan), and western regions (Inner Mongolia, Sichuan, Yunnan, Shaanxi, and Gansu) in mainland China.

Table S5. Percentiles of healthcare resources by years

	Number of healthcare institutions/ 10 000 population	Number of primary healthcare institutions/10 000 population	Number of equipment with a value of ⩾CNY 10 000 /1000 population*	Number of healthcare technicians/1000 population	Number of practicing (assistant) physicians/1000 population	Number of practicing physicians/1000 population	Number of registered nurses/1000 population	Number of beds/1000 population	Beds occupancy rate, %	Average length of stay, days
**2010**										
P5	3.51	3.09	4.93	3.11	2.52	2.14	1.07	2.88	70.80	9.4[illegible]
P95	11.51	11.06	22.82	5.96	3.77	3.54	3.43	4.73	96.10	13.0[illegible]
P95-P5	8.00	7.96	17.89	2.84	1.25	1.40	2.36	1.85	25.30	3.6[illegible]
**2015**										
P5	3.49	3.18	2.15	6.88	1.38	1.24	1.66	3.67	56.60	8.7[illegible]
P95	11.56	11.06	11.86	9.32	2.46	2.33	2.92	5.86	82.20	11.1[illegible]
P95-P5	8.07	7.88	9.72	2.44	1.09	1.09	1.25	2.19	25.60	2.4[illegible]
**2021**										
P5	4.29	3.93	1.25	4.47	1.62	1.11	3.02	5.00	76.00	8.3[illegible]
P95	11.84	11.42	8.74	6.65	2.55	2.11	4.17	8.04	96.10	10.4[illegible]
P95-P5	7.55	7.49	7.49	2.18	0.93	1.01	1.15	3.04	20.10	2.1[illegible]

P95-P5 was calculated by subtracting the 5th percentile from the 95th percentile.

*Including 15 provinces across eastern (Beijing, Liaoning, Jiangsu, Fujian, Shandong, and Guangdong)), central (Heilongjiang, Jiangxi, Hubei, and Hunan), and western regions (Inner Mongolia, Sichuan, Yunnan, Shaanxi, and Gansu) in mainland China.

Table S6. The distribution of healthcare resources across GDP tertiles and urbanization tertiles in mainland China in 2021

	GDP			Urbanization		
	Low	Middle	High	Low	Middle	High
**Healthcare infrastructure resources**						
Number of healthcare institutions/10 000 population	6.65	7.36	8.55	5.47	7.68	8.98
Number of primary healthcare institutions/10 000 population	6.32	6.87	8.13	5.13	7.29	8.57
Number of equipment with a value of ≥CNY 10 000 /1000 population*	7.16	7.26	8.34	6.56	7.09	8.53
**Healthcare human resources**						
Number of healthcare technicians/1000 population	7.75	8.19	8.31	7.76	8.05	8.10
Number of practicing (assistant) physicians/1000 population	2.99	3.09	3.11	2.86	3.12	3.16
Number of practicing physicians/1000 population	2.49	2.66	2.60	2.31	2.61	2.73
Number of registered nurses/1000 population	3.49	3.63	3.66	3.53	3.54	3.60
**Healthcare service resources**						
Number of beds/1000 population	6.55	6.77	7.13	5.90	6.88	7.32
Number of outpatient visits in healthcare institutions	1111792429	2189502572	5170738435	2242465206	2796948745	3432619485
Number of outpatient visits in primary healthcare institutions	45160	102351	277512	114120	150352	160551

*Including 15 provinces across eastern (Beijing, Liaoning, Jiangsu, Fujian, Shandong, and Guangdong)), central (Heilongjiang, Jiangxi, Hubei, and Hunan), and western regions (Inner Mongolia, Sichuan, Yunnan, Shaanxi, and Gansu) in mainland China.

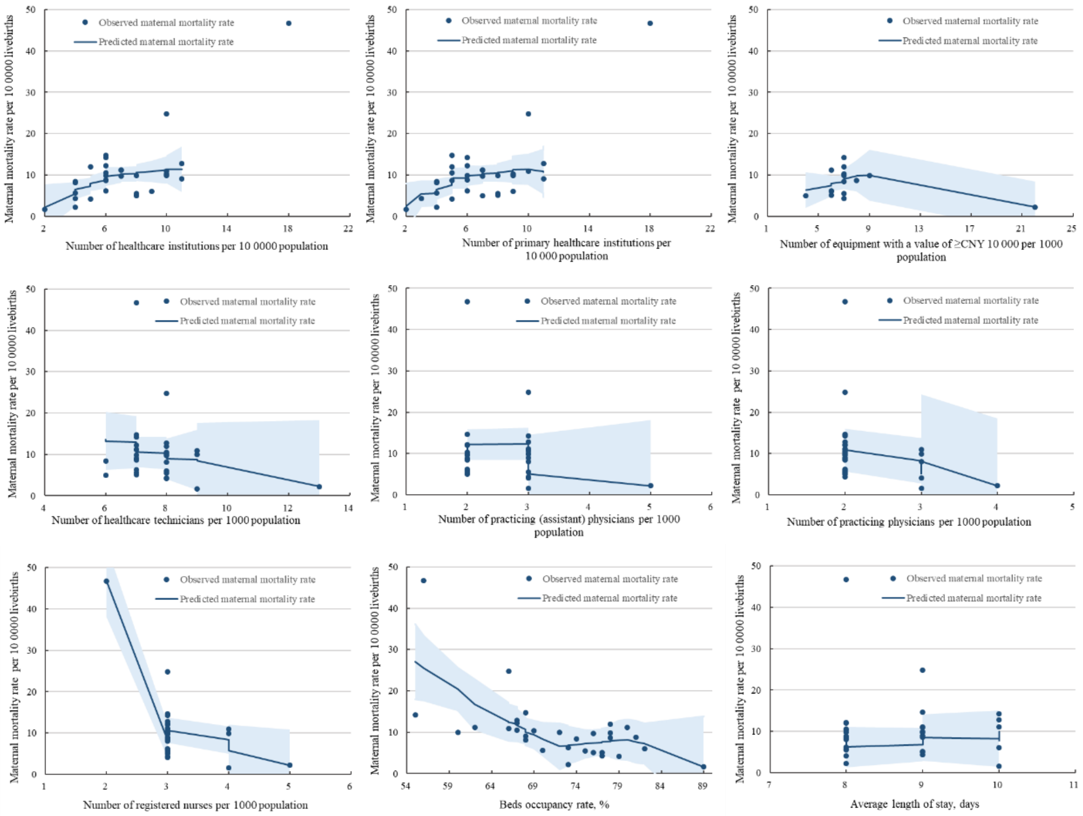

Figure S1. Relation between maternal mortality rate (per 10 0000 livebirths) and density of healthcare resources in 2021 for 31 provinces in mainland China. The dark blue point indicates observed maternal mortality rate, and the blue shade indicates 95%CIs. The curve was fit to the data by spline regression models.

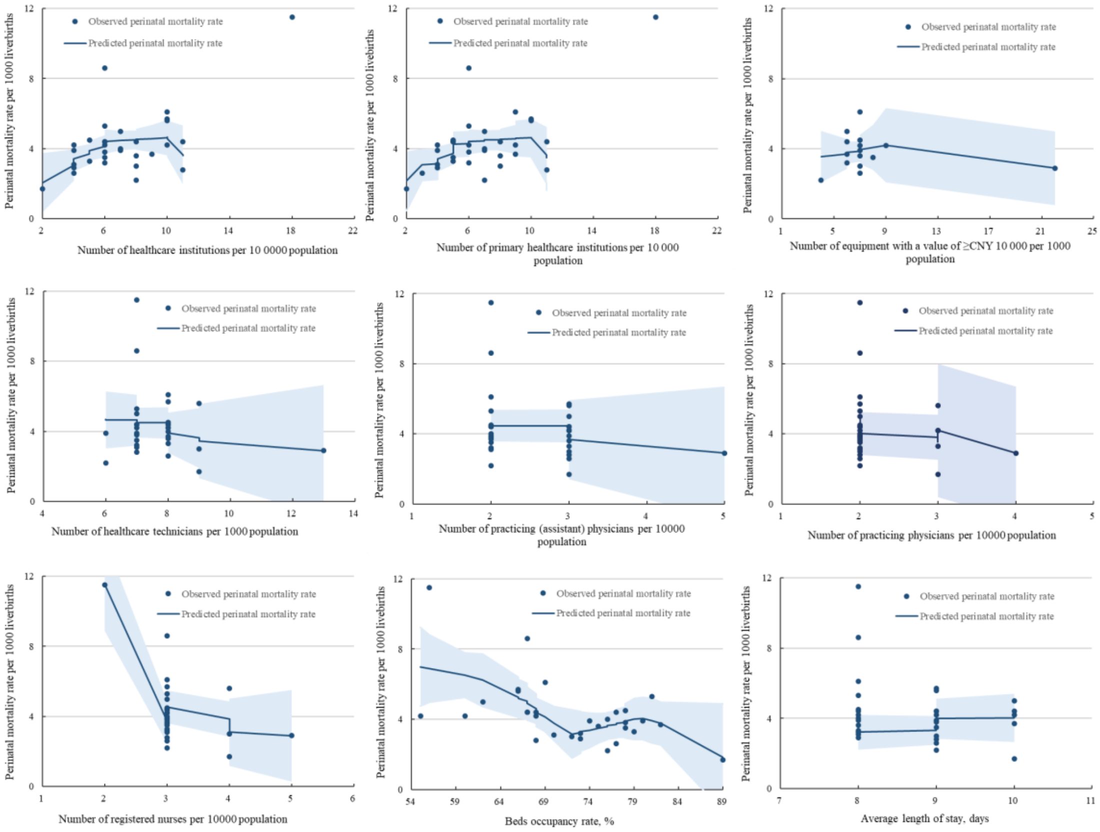

Figure S2. Relation between perinatal mortality rate (per 1000 livebirths) and density of healthcare resources in 2021 for 31 provinces in mainland China. The dark blue point indicates observed perinatal mortality rate, and the blue shade indicates 95%CIs. The curve was fit to the data by spline regression models.

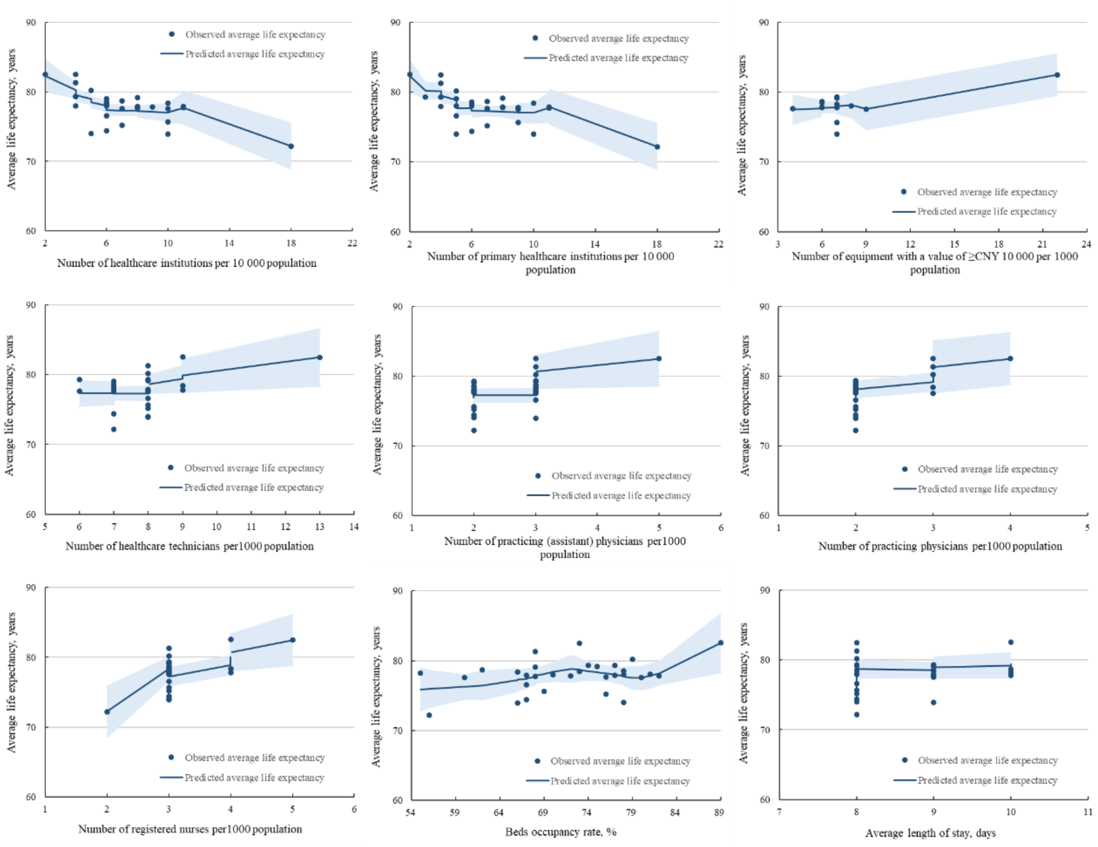

Figure S3. Relation between average life expectancy (years) and density of healthcare resources in 2021 for 31 provinces in mainland China. The dark blue point indicates observed average life expectancy, and the blue shade indicates 95%CIs. The curve was fit to the data by spline regression models.

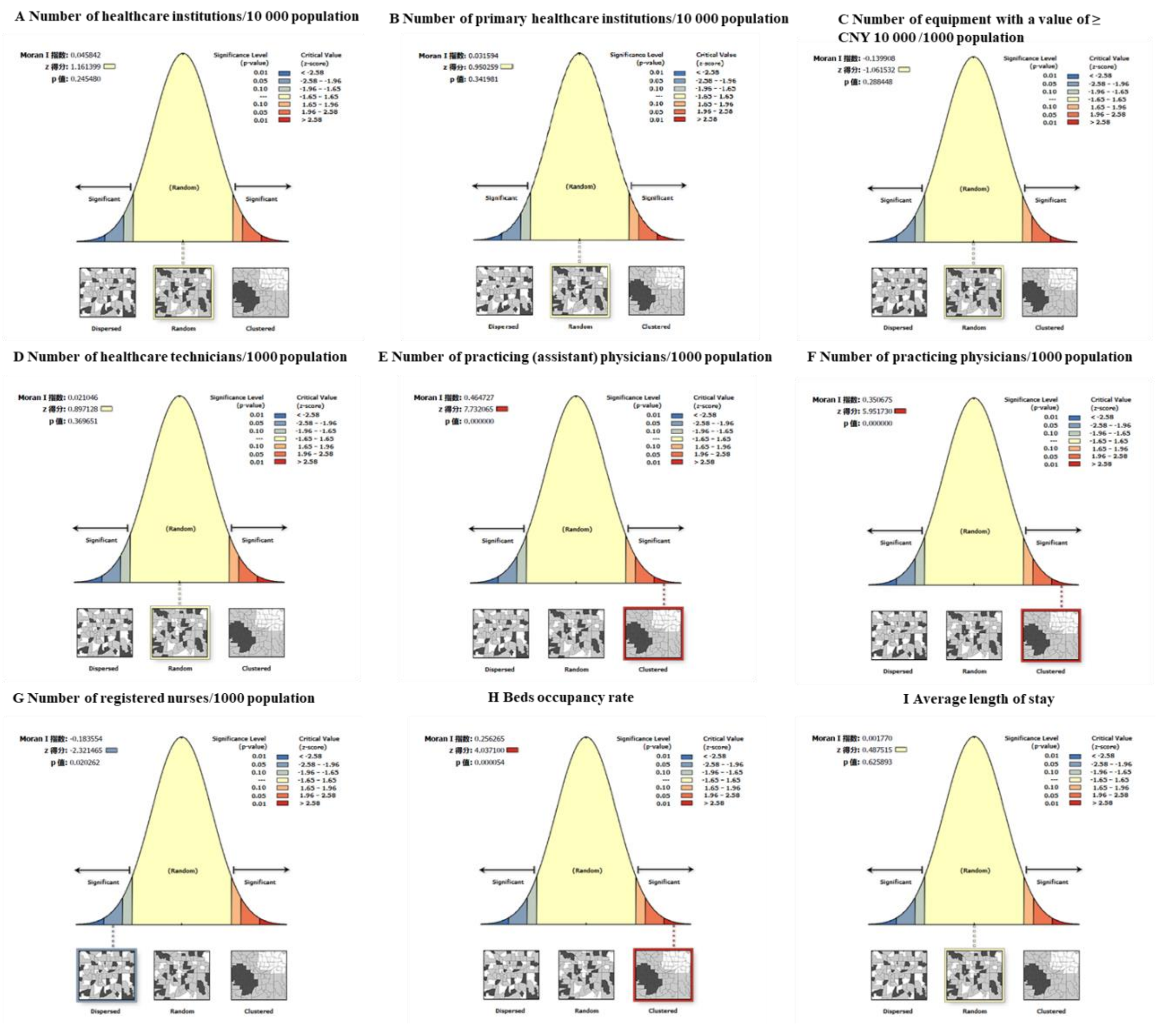

Figure S4. Global spatial autocorrelation analysis for healthcare resources.

Global spatial autocorrelation analysis for number of healthcare institutions/10 000 population (A), number of primary healthcare institutions/10 000 population (B), number of equipment with a value of ≥CNY 10 000/1000 (C), number of healthcare technicians/1000 population (D), number of practicing (assistant) physicians/1000 population (E), number of practicing physicians/1000 population (F), number of registered nurses/1000 population (G), beds occupancy rate (H), and average length of stay (I).
